# Debriefing Gold: Harnessing the Power of Debriefing Data to Inform Education

**DOI:** 10.5811/westjem.2022.12.57727

**Published:** 2023-01-11

**Authors:** Alexander Meshel, Barbara Dilos, Lillian Wong, Daniel Lugassy, Suzanne Bentley

**Affiliations:** * The Mount Sinai Hospital, Department of Anesthesiology, Perioperative and Pain Medicine, New York, New York; † NYC H+H/Elmhurst, Department of Anesthesiology, Elmhurst, New York; ‡ Icahn School of Medicine at Mount Sinai, Department of Anesthesiology, Perioperative and Pain Medicine, New York, New York; § Icahn School of Medicine at Mount Sinai, NYC H+H/Elmhurst, Department of Emergency Medicine, New York, New York; || NYC H+H/Elmhurst, Simulation Center, Elmhurst, New York; # Icahn School of Medicine, Department of Emergency Medicine, New York, New York

## Abstract

Debriefing is a critical element in healthcare, both in the clinical environment and in the simulation lab. Often, what is said at a debriefing is not recorded, leading to loss of critical data that could be used to inform future simulations, education, and systems improvement. In this perspective piece, we explain the powerful role that capturing debriefing data can have for identifying themes to improve learners’ knowledge and skills, as well as inform data-driven systems change and initiatives.

## INTRODUCTION

Debriefing in healthcare is an interactive, bidirectional, and reflective discussion regarding a recent event. 1 It requires some form of facilitation to enhance the resulting reflection. 2 It allows learners to critically evaluate their own clinical performance to better learn through the analysis of their actions, thoughts, and emotions. 2–4 Different tools have been developed to aid in debriefing,^5^–^7^ with entire publications concluding that there is no “one right way” to debrief.^8^ Furthermore, debriefings can occur at varying time points surrounding an event and can be self-directed or facilitator-led.^9^–^11^

Educators commonly incorporate standardized teaching points within debriefings related to the session’s educational objectives. In the simulation center, group debriefings often rely on notes scribed by the facilitator (such as on a white board). The educator may scribe debriefing points inclusive of best practices, new knowledge, and ideas for improvement—the golden nuggets of education and innovation to be gleaned. Following the debriefing, however, these white boards are often erased as the facilitator moves to the next group of participants and this “debriefing gold” is “lost.” The learner has hopefully received valuable, new knowledge; however, without systematic recording of debriefing discussion points, there is a huge missed opportunity for building a data-driven system to help inform future educational lessons, materials, and simulations and to assess for recurring patterns or themes elucidating potential systems issues or latent safety threats (LST).

## VALUE OF DEBRIEFING DATA

We have implemented a system in which debriefing discussions in the simulation center and after in situ simulations are recorded in a database. Following in situ simulations, debriefings are the time when participants identify specific lessons learned, positive occurrences to reinforce, areas of opportunity, and LSTs. During our debriefings, we use a white board or giant pad to visually operationalize the discussion of what went well and what could have gone better during the simulation and why. These comments and discussion points are then transcribed into the database, where they are further delineated and categorized by the debriefing phase during which the topic discussion arose (eg, reactions, analysis, summary phase), whether they represent a positive or negative/area of opportunity or an LST. The LSTs were then categorized as equipment, medication, technical skill/knowledge, or a systems issue. Finally, action items and their remediation and generated outcomes were recorded. We use the debriefing system in this instance to capture the LSTs in order to escalate and mitigate them (Table 1). We ensure that we close the loop by reporting outcomes back to the participants, fostering buy-in to simulation as a change agent, particularly when conducting future in situ simulations. Over time, this data may be thematically coded and compared to real-case outcomes or incidents to create a data-driven approach to capture emerging themes and analyze consistencies or inconsistencies across cases.

Additionally, we use debriefing data to inform educational activities. While the gold standard is to create simulation curricula based on formal needs assessment, in reality many simulations are also developed based on leadership, clinician, or simulation educators’ clinical “feeling” of what they think is most needed educationally or at times from a single, root cause analysis (RCA) outcome. Simulation can be an effective tool for high-acuity, low-frequency events; however, simulation may not always be the highest yield solution and it may risk leading to a large opportunity cost in investing time and resources in education that may be better served by a less resource-intensive educational modality. These rare occurrences can result in numerous fallacies and biases based on perception of both severity and frequency of event and lead to perhaps misguided investment in simulation to address such issues. We should acknowledge that not everything can or should be simulated and advocate that educational modalities and investments should be conducted via a strategy that is as highly informed as possible. If simulation is selected as the appropriate corrective action from a RCA, for example, capturing and analyzing debriefing points may provide greater insight into both participant action(s) and knowledge, as well as a systems assessment of equipment, resources, and educational efforts.

Our database has allowed for iterative expansion of educational modalities based on data captured during ongoing simulations and serves as a robust and evolving needs assessment. While doing an extensive in situ simulation initiative on cardiac arrest focusing on identifying and mitigating LSTs, debriefings identified numerous deficiencies in the team leader’s performance with evident recurring downstream effects.^12^ In more than 90% of the simulations, we found there to be at least one debriefing point relating to a deficiency or area of improvement in the team leader performance. We therefore developed a new cardiac arrest team leader training specifically addressing the objectively highest frequency debriefing trends seen in leader performance from the database. Upon implementation of this new program, the learners report that they are now being taught about and given the opportunity to practice the specific problematic areas that they continually encounter but have been unsuccessful in rectifying on their own. By capturing debriefing points over time, we were able to create cases that our learners found particularly high fidelity to their day-to-day work and realistic of the problems they encounter in their clinical performance and teamwork.

A debriefing data system not only allows for educational initiatives expansion and creation but can target workforce well-being and be used for the development and inclusion of other resources. From the analysis of the reactions phase discussions, many participants had noted high stress from particular simulation case types. The capture of this specific data theme allowed us to use this information to ensure we brought and provided tailored emotional support resources to these simulations (which we now provide to all participants in all simulations). We were able to inform facilitators about prior reactions and allow them to be on alert for participants noting particularly strong reactions or distress to the simulation. For example, many participants noted strong negative reactions following certain trauma simulations. In discussions it appears this was due to the severity of the case, as well as unique previous experiences with similar scenarios in real cases. We have also since worked to alter our pre-brief to ensure a psychological space that is as maximally safe as possible, and we have brought emotional support service resources to future simulations that participants could use to discuss the simulation or other clinical situations.

This method of data capture can be used across a wide variety of simulation initiatives. While we have used it mainly during both in situ and simulation lab sessions, it can also be used during post-event clinical debriefing by clinical faculty. We plan on implementing this system for data capture with clinical faculty leading post-event debriefing to record similar information that can then be analyzed across events. This will further allow the simulation faculty and leadership of the respective departments who are using our system to understand trends and themes, allowing for design of simulations centered directly on the needs or issues identified in real clinical events. Lastly, this system can be applied to other simulation environments including debriefings of procedures learned on task-trainers and telesimulation.

There are some limitations and barriers to implementation. For example, a single simulation facilitator would classify and code debriefing points based on best judgment (unless the simulations were part of a specific research initiative with two reviewers). While this may introduce bias in the capture, it allows for logistically easier implementation to elicit patterns of performance (both strengths and weaknesses). There are also barriers to implementation including buy-in from departmental and hospital leadership regarding how debriefing data would influence their educational and simulation efforts, as well as the simulation faculty’s time required to create and maintain this system. We do believe, however, that the outcomes of maintaining a database of debriefing points offer data-driven approaches to help inform new initiatives, education, and future simulations.

## CONCLUSION

Clinicians and educators should recognize the inherent power of the debriefings they lead and the information “gold” gained through discussing learners’ reactions, case analysis, and reasoning between positives and areas of opportunity in the cases. We educators are in a unique position to leverage observed, data-driven patterns to construct thoughtful, deliberate, and timely future programming. Given the time constraints of educators and learners of all levels, it is crucial that we use many different metrics, data points, and strategies to derive our education activities and maximize their fidelity and utility to local learner environments and phenomena. Every white board that is erased at the conclusion of a debriefing is a missed opportunity, as we simply erase the gold nuggets of gleaned information that could instead inform future data-driven programs. Capturing debriefing data in a systematic way for use as an ongoing needs assessment is a powerful method to further operationalize and inform what we can and should be teaching.

## Figures and Tables

**Table 1:** Debriefing data outcomes recorded and examples.

Topic discussed	Debriefing phase (eg, reactions, analysis, summary)	Positive or negative/opportunity	LST (yes/no)	LST category type (equipment, medication, technical skill/knowledge, systems issue)	Outcome
Delay in transfusion due to assigned staff “runner” not aware of location of blood bank	Analysis	Opportunity	Yes	Systems issue; knowledge gap	Cognitive aid/map creation; education of team
Role confusion on trauma team	Analysis	Opportunity	Yes	Systems issue	Trauma team guide with roles and infographic creation; education of team through future simulations targeting roles
Inability to locate rarely used equipment (pediatric tray), with participants noting resulting “high stress” from situation	Reaction; Analysis	Negative/Opportunity	Yes	Equipment	Equipment location review and redesign; simulation participants informed of resulting changes; teams educated; wellness resources provided

*LST*, latent safety threat.
